# Subglottic Stenosis in Children: Preliminary Experience from a Tertiary Care Hospital

**DOI:** 10.1155/2020/6383568

**Published:** 2020-12-15

**Authors:** Manzoor Ahmad Latoo, Aleena Shafi Jallu

**Affiliations:** Otorhinolaryngology, Head & Neck Surgery, Government Medical College Srinagar, Srinagar, Jammu & Kashmir, India

## Abstract

**Introduction:**

This retrospective study describes our experience in the evaluation and management of infants with subglottic stenosis.

**Materials and Methods:**

The study included 10 patients aged between 1 wk and 18 months with 6 cases having congenital subglottic stenosis and 4 cases having acquired subglottic stenosis.

**Results:**

6 patients had grade I, 3 patients had grade II, and 1 patient had grade III subglottic stenosis. Tracheostomy was required in 4 patients at the time of presentation. 7 patients were treated successfully with Bougie dilation followed by topical application of mitomycin, whereas 1 patient who failed to serial dilation needed open reconstructive procedure. Laser excision of the anterior subglottic web was performed in one patient. Another patient with underlying cerebral palsy could not be operated upon and was managed with tracheostomy.

**Conclusion:**

Subglottic stenosis may be effectively man-aged with endoscopic surgical techniques, although the number of such sittings required varies with the type and severity of stenosis. Open surgical procedures need to be individualised as per the needs of the patient only after all the other endoscopic possibilities have been exhausted.

## 1. Introduction

Subglottic stenosis (SGS) is a narrowing of the subglottic airway, which is housed in the cricoid cartilage, i.e., below the glottis and above the first tracheal ring [1]. This can be congenital or acquired. Congenital subglottic stenosis (SGS) has several abnormal shapes. It occurs as a part of congenital genetic syndromes and is considered the third most common cause of stridor in this period of life after laryngomalacia and vocal cord palsy [2]. Congenital subglottic stenosis may be classified as membranous or cartilaginous. The membranous is the most common and mildest of congenital stenosis. Membranous SGS is usually circumferential, soft, and dilatable. In contrast, the cartilaginous SGS has a variable appearance, including mild normal shape with narrowed lumen, or can have abnormal shape of cricoid cartilage with prominent lateral shelves giving an elliptical appearance of lumen. 

More than 90% of cases of SGS are secondary to endotracheal intubation [3, 4]. Acquired SGS (ASGS) is the most common acquired anomaly of the larynx in the paediatric age group and is the most common abnormality necessitating tracheotomy in children below one year of age [5]. The structures of the infant larynx are more pliable and less fibrous, making the infant airway more susceptible to narrowing edema and less easily palpable. One millimetre of decrease circumferentially in the subglottis reduces the cross-sectional area by 60%.

The standard methods for airway evaluation is direct laryngoscopy and direct bronchoscopy, while surgical therapies for subglottic stenosis (SGS) include serial endoscopic dilation, open reconstruction, anterior cricoid split (ACS), single-stage laryngotracheoplasty with cartilage expansion, anterior and posterior cricoid splitting with costal cartilage grafts placed anteriorly and posteriorly, and partial cricotracheal resection [1]. Other investigations include imaging of the airway (airway films, CT scan, etc.), and in older stable children, pulmonary function tests may be of value.

Endoscopic treatments include dilations with pneumatic balloons or rigid dilators, laser incisions, or cold scalpel [6]. Until the 1980s, endoscopic dilations were most often used to treat subglottic stenosis. However, surgical reconstruction began to boom after the publication of Fearon and Cotton [7]. These are invasive and time-consuming surgeries, with hospitalization in intensive care units, morbidity, and even mortality. Some of these patients will even require postsurgical endoscopic treatment to improve results [6]. Endoscopic dilations were indicated for stenosis grades I and II and in those SGS recurrences in postsurgical patients with laryngotracheal reconstructions [8]. Currently, patients with SGS grade III are also candidates for dilations [4].

Topical substances such as steroids, anticoagulants, and mitomycin C (MMC) have been used to optimize endoscopic treatment. Although adjuvant treatments such as steroids and antibiotics have been investigated, much attention in recent years has turned to the use of topical mitomycin C (MMC). Mitomycin, an antibiotic from *Streptomyces caespitosus*, has antifibrinogenic and antineoplastic activities that have been shown to block fibroblast proliferation and thereby reduce scar formation on topical application. The use of MMC in the treatment of airway stenosis was first reported in 1998 and is now routinely used in the endoscopic management of laryngotracheal stenosis [9].

## 2. Materials and Methods

The study included patients referred from neonatal/paediatric tertiary care hospitals of the valley who were diagnosed to have subglottic stenosis between 2015 and 2018. All the cases were studied in detail and data were obtained with regards to the age of presentation, mode of presentation, need for tracheostomy, history of intubation, gender, findings on fiber-optic laryngoscopy and rigid endoscopy, classification and grade of stenosis, any comorbidities, procedure performed, and the eventual outcome.

All the patients underwent direct laryngoscopic and bronchoscopic examination under general anesthesia, and further management was dictated by the grade and type of stenosis. The stenosis was graded as per the Cotton–Myer classification (grade I: 0–50%, grade II: 51–70%, grade III: 71–99%, and grade IV: no detectable lumen), using the size of the endotracheal tube passed as a guide . Dilation was performed with rigid (bougie) dilators with increasing diameters until the patient's age-corresponding diameter was reached. The result of the expansion was then evaluated with the same technique. All the diagnostic and therapeutic procedures were carried out by the same surgeon who is a professor by designation.

## 3. Results

A total of 10 patients aged between 1 wk and 18 months were included in the study with the mean age of presentation being 3 months. There were 6 male (60%) and 4 female (40%) patients. 4 patients presented with stridor, 4 more patients had stridor on exertion, and 2 patients had presented with repeated desaturation (Table 1). All the patients had undergone endoscopic laryngeal examination before being referred to the department of Otorhinolaryngology, Head & Neck Surgeryfor definitive treatment. Tracheostomy was required in 4 patients (40%) at the time of presentation. These included 2 patients with acquired subglottic stenosis (ASGS) and 2 patients with congenital subglottic stenosis (CSG).

 Based on the Cotton-Myer classification, 6 patients had grade I stenosis, 3 patients had grade II stenosis, and 1 patient had grade III stenosis (Table 2).

**Figure 1:**
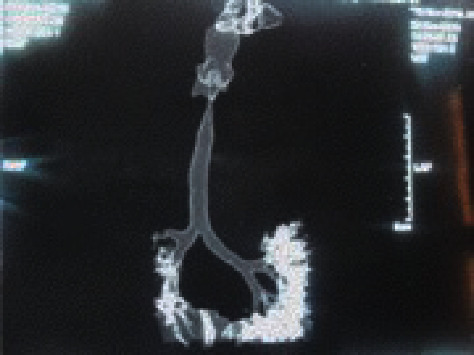
Subglottic stenosis with 3D reconstruction on a multidetector CT in an 18-month-old child with aquired subglottic stenosis.

**Figure 2:**
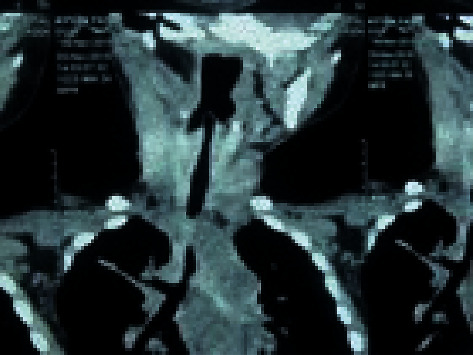
Preop coronal CT appearance in a child with acquired subglottic stenosis.

**Figure 3:**
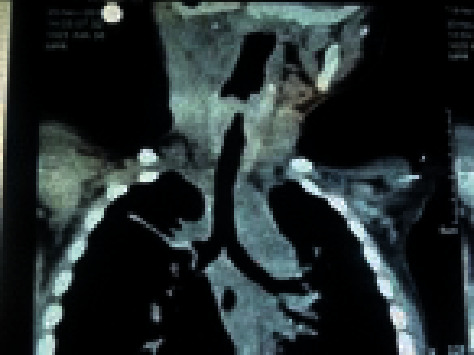
Postoperarative appearance on coronal CT images, after surgery for acquired subglottic stenosis; note the adequacy of the airway.

Four patients (40%) had a history of intubation prior to development of symptoms and were found to have subglottic stenosis on fiberoptic laryngoscopic (FOL) examination and labeled as acquired subglottic stenosis. Two patients with acquired subglottic stenosis were born prematuraly with a mean gestational age of 28 wks and were intubated for the same. Both these patients developed stridor immediately after extubation. One of the patients with acquired subglottic stenosis had developed neonatal septicemia and remained intubated for 6 weeks. The patient presented with repeated episodes of desaturation which were aggravated by upper respiratory tract infection (URTI). The patient had developed fibrosis and granulations predominantly posteriorly and was tracheostomised. Another patient with acquired subglottic stenosis presented at the age of 18 months when he had undergone repeated intubations for multiple surgeries for congenital vesicourinary anomalies (Figures 1–3) and needed tracheostomy at the time of presentation. All the patients with acquired subglottic stenosis had fibrous subglottic stenosis with grade I in two patients and grade II in two patients.

Six patients (60%) had no history of prior intubation, and were labeled as congenital subglottic stenosis, the youngest being 1 wk old and the oldest being 4 months old. Two of these presented with stridor, 3 had stridor on exertion, and 1 patient had presented with repeated desaturation aggravated by inflammatory insult. Two patients of congenital subglottic stenosis were found to have cartilaginous stenosis and required tracheostomy, whereas the other 4 had membranous subglottic stenosis.

**Figure 4:**
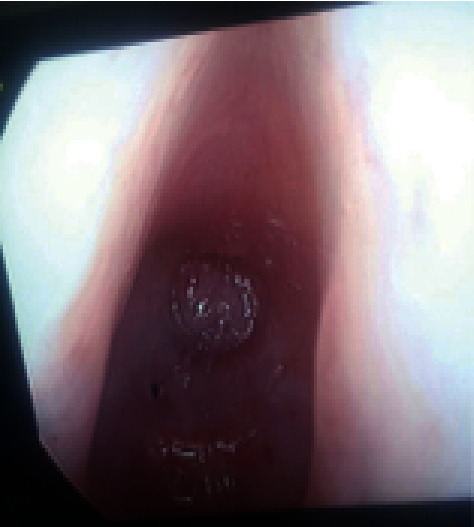
Grade III membranous subglottic stenosis with only a pinpoint opening as seen on endoscopic examination.

**Figure 5:**
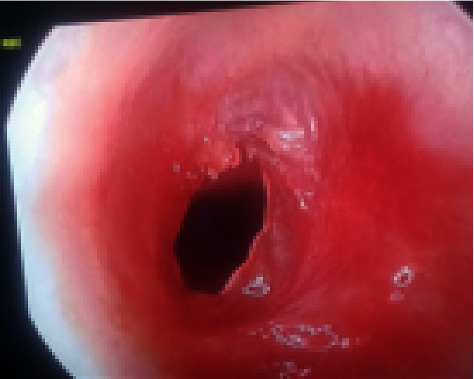
Postoperative endoscopic view of grade III subglottic stenosis after first sitting of bougie dilation.

**Figure 6:**
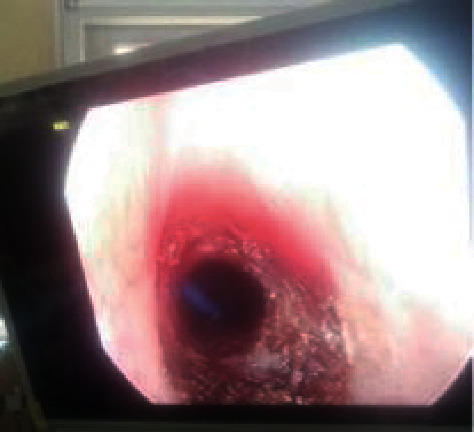
Postoperative picture after third sitting of bougie dilation for grade III subglottic stenosis.

**Figure 7:**
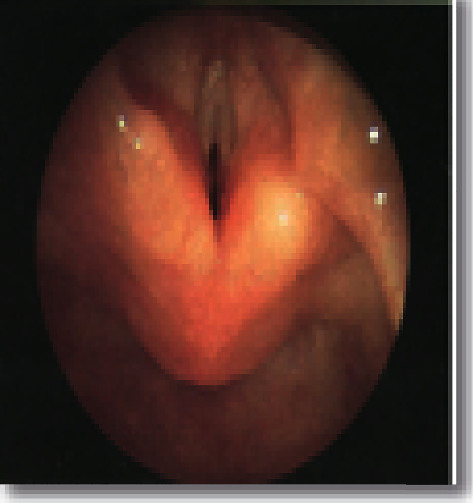
Anterior subglottic web.

**Figure 8:**
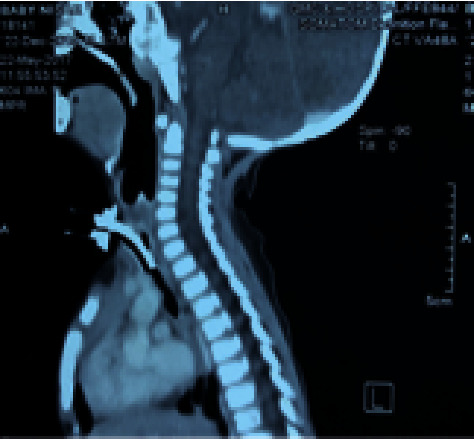
Preoperative sagittal CT image with tracheostomy tube in situ.

**Figure 9:**
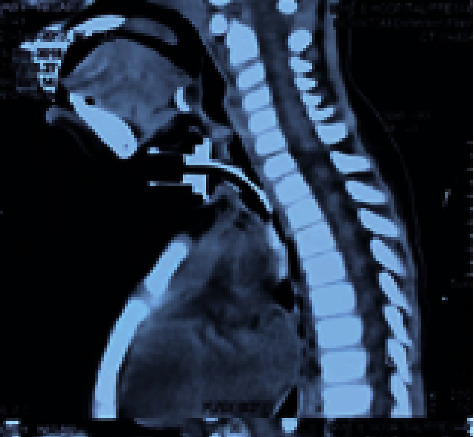
Postoperative CT image showing adequate subglottic airway lumen.

Bougie dilation followed by topical application of mitomycin was performed in 8 patients (80%). An initial evaluation was performed by rigid bronchoscopy. Subsequently, dilation of the stenotic area was performed and was followed by topical application of mitomycin C at a concentration of 0.5 mg/mL for 2 min. At least two sittings of rigid bronchoscopy at an interval of 6 wks were performed on these patients. 4 patients had obtained adequate subglottic lumen on second look and did not require any further intervention. These patients were closely followed for 2 yrs postoperatively before declaring them as treated successfully. 2 patients required second stage dilation, whereas another one required 3 stages of dilation plus mitomycin application (Figures 4–6). One patient with congenital subglottic stenosis had a membranous web (Figure 7) anteriorly which was excised using CO_2_ laser followed by mitomycin C application and had completely healed and epithelialised at second look. Another patient of congenital stenosis who presented at 4 months had underlying cerebral palsy and was managed conservatively with tracheostomy in place (Table 3). One among two other tracheostomised patients was decannulated successfully after serial dilations (Figures 8 and 9), whereas the other one with h/o neonatal sepsis had developed tracheomalacia and was decannulated at the age of one year. One of the patients of congenital subglottic stenosis with grade II cartilaginous stenosis had failed to respond to dilation and was planned for open procedure and could not be decannulated (Table 4) but the patient was lost to follow-up. Hence, a decannulation rate of 50% was achieved.

##  4. Discussion

In the early 20th century, subglottic stenosis was somewhat uncommon. The figures from the literature showed a considerably varied prevalence of 1%–8% [10, 11]. Since the 1960s, studies have reported an increase in the incidence of acquired SGS, with the age at presentation shifting toward young infants. Postintubation stridor is a common problem in the paediatric intensive care setting (over 44% in the current article by Schweiger et al. [12]). Yet, the incidence of airway complications associated with intubation is relatively low. A recent prospective study reported that an incidence of postintubation subglottic stenosis in children was 11.38% [13]. This can be attributed to improved ventilation methods for neonates in intensive care units, while saving lives of children with bronchopulmonary dysplasia, hyaline membrane disease, or neonatal septicemia requiring prolonged periods of endotracheal intubation. The posterior glottis bears the maximum brunt of injury because of the posterior displacement of the endotracheal tube at the base of the tongue and posterior angulation of the trachea. This explains the synchronous involvement of the posterior glottis and of the subglottis in some cases of prolonged intubation [14]. Subsequent progress in ventilatory techniques and the widespread use of artificial surfactants have led to a decrease in the duration of intubation and even its complete avoidance, thereby reducing the incidence of acquired stenosis. A new prospective study even implied that undersedation might be a risk factor for the development of subglottic stenosis in intubated children [15]. A key concept that has emerged is that the appropriate size of the endotracheal tube is not that of the age-appropriate tube, but rather the child-appropriate endotracheal tube.

Medical advances have however, had little impact on the incidence of congenital stenosis [10, 16–18]. In our study, a predominance (60%) of congenital stenosis was observed and acquired stenosis in all 4 cases was due to prolonged intubation. In all cases, the diameter of stenosis was estimated using endotracheal tubes that could be passed through the stenotic segment during microlaryngoscopy and bronchoscopy (MLB), and the severity was objectively scaled using the Myer–Cotton Grading System, based on endotracheal tube sizing [19]. On radiographic imaging, these patients have classic hourglass subglottis narrowing of the air column.

A simple, effective, minimally invasive therapy could be of great benefit for the treatment of this pathology. The option of dilating the narrow airway is one of them. Balloon dilations have been used with good results [8, 19–23]. However, there are few reports of dilations with rigid or semirigid dilators [21, 24]. Despite the increase in the implementation of treatment with dilations, no selection criteria have yet been established to optimize the results [25]. The success rate of endoscopic treatment has varied according to the reports and the method used, ranging from 60% to 70% by balloon dilations [20, 23]. Up to 100% reported with rigid dilations although this includes a large percentage of patients with stenosis grade 1 and a high number of procedures over a long period [21]. In our case series, the result was favorable in 87.5% of the patients treated. Hence, we feel this treatment is effective to all patients with subglottic stenosis grades I to III.

In one recent study, it was concluded that SGS treatment using rigid dilators under endoscopic control is a minimally invasive and effective method [26]. Dilation with rigid dilators could be indicated as first-line treatment for SGS grades 1–3. They recommended 3 endoscopic dilation procedures and then, in the absence of response, consideration of other treatment alternatives. Given that the patients who did not respond were subsequently operated on without any difficulty attributable to the dilations, we think that the initial endoscopic treatment does not interfere with future surgical treatment in the event of failure.

Laser is mostly used for ablation of granulation tissue and laryngeal or subglottic webs. Treatment is based on dilation of the stenotic area; however, wound healing can result in restenosis. Drugs that prevent fibroblast proliferation can slow down or even inhibit this process. Mitomycin is a natural antibiotic produced by *Streptomyces caespitosus*. In 1958, Wakaki et al. described mitomycin C (MMC) [27]. In addition to being an antibiotic, mitomycin C acts as an antineoplastic or alkylating agent by inhibiting DNA synthesis. Numerous studies have shown that mitomycin inhibits fibroblast and scar formation in vitro as well as in vivo [28, 29]. MMC was first used in the airways in the late 20th century, and since then, few studies have demonstrated its efficacy [30, 31], whereas others have shown it to be ineffective [32]. Previous studies have shown that MMC appears to be effective in the treatment of postoperative stenosis [33]. Mitomycin application has been associated with an extended time interval between endoscopic treatments [34]. In our study, mitomycin application was performed in all patients. It was used in conjunction with bougie dilation and CO_2_ laser lysis of laryngeal web. There was a significant reduction of granulation tissue after mitomycin application. Patients with grade I or grade II stenosis and membranous grade III were managed conservatively with endoscopic procedures. The mean number of endoscopic procedures performed per patient increased with the increasing severity of stenosis. An open reconstructive procedure was considered in only in 1 patient, a case with grade II cartilaginous stenosis that showed no improvement with conservative endoscopic management. The patient had a failed decannulation attempt due to stridor and respiratory distress. When the draft for this study was being compiled, the patient was on tracheostomy and was due for open reconstructive procedure. Many authors have concurred that cricotracheal resection (CTR) is acceptable, and even preferred, for high-grade subglottic stenosis, with the largest reported series of paediatric cricotracheal resection [35] achieving longterm decannulation rates of more than 90%. In this study, open procedure was indicated only in one case of grade II cartilaginous stenosis.

In conclusion, experience in airway endoscopy is crucial in the diagnosis and management of paediatric subglottic stenosis. When these children present, it is important to perform a thorough history and physical examination. The physical exam should include a thorough head and neck exam as well as characterisation of stridor and signs of respiratory distress. A flexible laryngoscopic examination should be performed. The important things to document during endoscopy include the outer diameter of the largest bronchoscope or endotracheal tube that can be passed through the stenotic segment, the location and length of the stenosis, other separate sites of stenosis, and reflux changes. After removing the sizing endotracheal tube or bronchoscope, it is important to observe the stenotic segment for edema which may result in the need for tracheostomy. Children with SGS are subjected to prolonged periods in hospital and often require multidisciplinary approaches, with the head and neck surgeon taking a primary role. Tracheostomy is often required. Conservative endoscopic dilation alone may be sufficient in the majority of grade I, grade II, and even in grade III stenosis. More severe cases might warrant reconstructive surgery. LTR (laryngotracheal reconstruction) remains the intervention of choice, with CTR being a complement where indicated, such as in failure despite repeat LTR [36].

With the decreasing incidence of acquired SGS due to improved medical care, major open surgical cases and experiences are now rarely encountered. Surgical intervention should not be rushed into until after all possibilities have been carefully considered.

Small sample was a limitation to the present study, but with the rarity and decreasing prevalence of SGS, it may be difficult to design an accurately powered prospective study. Small sample was a limitation to the present study. Nonetheless, our study does provide some insight into the current management techniques and outcomes of SGS which in our hands have been good.

## Figures and Tables

**Table 1:** Presenting complaints.

Symptoms	*n* (10)	Percentage
Stridor	4	40
Stridor on exertion	4	40
Repeated desaturation	2	20

**Table 2:** Type and grade of subglottic stenosis.

	*n*	Percentage
*Type*		
Congenital subglottic stenosis	6	60
Acquired subglottic stenosis	4	40

*Grade*		
I	6	60
II	3	30
III	1	10

**Table 3:** Modes of surgical intervention performed.

Grade of stenosis	Bougie dilation with mitomycin M application	CO_2_ laser excision with mitomycin C application	Failed procedure	Tracheostomy only procedure	Total (*n* = 10)
Grade I	5	1	0		6
Grade II	2	0	1	1	3
Grade III	1	0	0		1

**Table 4:** Decannulation rate.

		Percentage
Tracheostomy required	*n* = 10
Yes	4	40
No	6	60

Patient decannulated successfully	*n* = 4
Yes	2	50
No	2	50

## Data Availability

The supporting data used are appended under reference. Some of these are open source articles, and others have been accessed through institutional licences.
